# Effects of Visual Working Memory on Individual Differences in Echolocation Performance in Sighted Participants

**DOI:** 10.1177/2041669519872223

**Published:** 2019-08-29

**Authors:** Tomoki Maezawa, Jun I. Kawahara

**Affiliations:** Department of Psychology, Hokkaido University, Sapporo, Japan

**Keywords:** echolocation, perception, visual spatial working memory

## Abstract

Echolocation performance differs widely among individuals. This study examined a possible factor that may explain this variation, namely, visual working memory, which is a subcomponent of spatial working memory. Sighted participants performed an object-detection task consisting of initial testing on 2 separate days (up to 8 days apart) with follow-up testing on a third day (up to 1 month after the second day of testing) while manipulating the target distance from 20 to 50 cm. Participants performed two types of visual spatial working memory tasks, one of which required them to memorize color–location combinations and the other, an imaginary pathway. The participants’ performance on the object-detection task generally improved in the first 2 days, but there were substantial individual differences in detection ability. A positive correlation was observed between performance on these tasks and visual working memory capacity, except on the second day, after detection ability had improved. These findings suggest that factors contributing to echolocation skill are related to nonauditory factors in a sighted group.

## Introduction

Echolocation is a method to localize objects and acquire object features (e.g., distance, size, shape, and surface of material) based on the reflection of sound. Echolocation ability has been extensively acknowledged in nonhuman species, such as odontocetes (e.g., Au, Branstetter, Benoit-Bird, & Kastelein, 2009) and some bats (e.g., Barclay & Brigham, 1994; Neuweiler, 1990). Similarly, humans with normal hearing can use echolocation (Rice & Feinstein, 1965; Schenkman & Nilsson, 2010). Particularly, the sighted population can echolocate (Teng & Whitney, 2011) using several types of echo-acoustic sounds, such as oral clicks and artificial click noises via loud speakers (Thaler & Castillo-Serrano, 2016; Tonelli, Brayda, & Gori, 2016; Tonelli, Campus, & Brayda, 2018), as well as auditory substitution to enhance spatial awareness for successful navigation (Kolarik, Scarfe, Moore, & Pardhan, 2016).

Echolocation skill can be acquired with suitable training, although most sighted groups are less sensitive to the cues available for echolocation (Kolarik, Cirstea, Pardhan, & Moore, 2014). Rapid improvements in echolocation performance occur in sighted populations following only a few training sessions (Teng & Whitney, 2011). These findings indicate the need for repetitive training to acquire echolocation skill. However, large individual differences in echolocation performance and the degree of improvement during echolocation training may complicate the establishment of systematic echolocation training protocols (e.g., Ekkel, van Lier, & Steenbergen, 2017), and some individuals are unable to acquire echolocation skills (e.g., Worchel, Mauney, & Andrew, 1950). Thus, a better understanding of the factors contributing to these individual differences is necessary to aid in the training of echolocation skills.

A plausible explanation for the individual differences is that echolocation performance is affected by variation in auditory sensitivity of the hearing system because performance is based on detecting and discriminating auditory echoic cues. A set of studies has supported this relationship (e.g., Carlson-Smith & Wiener, 1996; Schenkman & Nilsson, 2010). Specifically, experiences with echoic information, auditory perceptual learning, and task feedback are associated with echolocation performance (e.g., Teng & Whitney, 2011; Teng, Puri, & Whitney, 2012). However, the ability to discriminate echoic cues is not the only factor that explains the individual variability (Rice, 1967) because sighted populations, in particular, do not rely on echoic cues relative to blind populations. Thus, other factors are involved.

In this study, we argue that working memory capacity plays a role in echolocation performance. Working memory is the temporary storage and workspace to manipulate information for ongoing tasks (e.g., Baddeley, 2012; Ricker, AuBuchon, & Cowan, 2010), and functions in conjunction with a subsidiary slave system of the attentional control system, known as the central executive (Baddeley, 2000). The relevance of working memory in auditory cognitive processing has been acknowledged (e.g., Moossavi, Mehrkian, Lotfi, Faghihzadeh, & Sajedi, 2014). In fact, the degree of improvement in echolocation performance positively correlates with a test score for the Paced Auditory Serial Addition Task (PASAT; Gronwall, 1977), reflecting working memory and divided attention (Ekkel et al., 2017), in conjunction with auditory verbal aspects. The PASAT has been used as a measure of impairments in the central executive of working memory (e.g., Serino et al., 2007); thus, the results of Ekkel et al. (2017) support the notion that working memory capacity modulates echolocation performance in size discrimination. However, it should be noted that the individual differences in the ability to use echolocation was beyond the scope of Ekkel et al.’s study. Rather, their focus was on the differences in improvements in echolocation performance due to training. Moreover, even though the central executive component would contribute to the learning of size discrimination (Ekkel et al., 2017), the processing of echolocation relates to spatial perception; thus, the process should involve the spatial component of working memory. Thus, further investigation is required to examine whether echolocation performance is linked to the capacity of spatial working memory, rather than central executive functions.

Visual working memory (Luck & Vogel, 2013; Mance & Vogel, 2013) is a subsidiary system of working memory, contributing to maintenance of visual information to serve the needs of ongoing tasks. The concept of the spatial subcomponent of working memory has played a central role in explaining individual differences in cognitive performance in the spatial domain (Kyllonen & Christal, 1990). Given that high visual working memory capacity is predictive of higher performance on visual tasks, such as visual search, localization and detection of changes (e.g., Luria & Vogel, 2011; Unsworth, Fukuda, Awh, & Vogel, 2014), visual working memory should predict individual differences in cognitive performance involving spatial processes. Importantly, visual working memory capacity predicts individuals’ filtering efficiency, so that in individuals with high visual working memory capacity, irrelevant distractor-related information is efficiently excluded from storage in memory, thus preserving the capacity for purposeful use (Vogel, McCullough, & Machizawa, 2005).

Although the concept of visual working memory has been traditionally related to active maintenance of visual representations, recent studies on working memory have extended the concept to spatial processing of images in auditory modality tasks (Loomis, Klatzky, McHugh, & Giudice, 2012). We hypothesized that visual processing and experience would contribute to the representation of mental images and aid echolocation in sighted subjects. In fact, much effort has been expended on characterizing the relationship between echolocation and visual cognitive processing (Tao et al., 2015; Thaler & Foresteire, 2017; Thaler, Wilson, & Gee, 2014). Thus, we expected that sighted individuals with superior visual working memory capacity would perform better in auditory spatial tasks, such as target search and change detection tasks. In other words, individuals with high working memory capacity should be able to detect a target object from distractor noise due to high-filtering efficiency.

Thus, the aim of this study was to examine the association between echolocation performance in sighted individuals and how individual factors of visual working memory capacity contribute to performance. Specifically, we examined whether visual working memory capacity correlates with object detection performance in echolocation tasks. We also investigated whether improved detection performance is retained over a month, by asking participants to perform the same echolocation tasks 3 times over that time periods. Maintaining improved echolocation ability after training presents a challenge for echolocation learning. In one study (Zahorik, Bangayan, Sundareswaran, Wang, & Tam, 2006) that did not use an echolocation task, improved ability in sound localization persisted for at least 4 months. Similar retention of echolocation improvement was expected in this study.

We designed an object-detection task based on Schenkman and Nilsson (2010), whose participants determined the presence or absence of a target in front of them using echolocation, while the distance to the target was manipulated. The experiment lasted 120 trials (approximately 50 minutes) per day across 2 days and the amount of training during the task was greatly reduced from that used by Schenkman and Nilsson (2010), who used 56 trials per session across 36 sessions (2.5–3 hours) with feedback on every trial. We also omitted the feedback. Specifically, our experiment consisted of initial testing on 2 separate days (up to 8 days apart) with a follow-up testing on a third day (up to 1 month apart). Two sighted participants in our pilot study showed a chance level in detecting a target placed more than 50 cm away; thus, we chose a target distance of 20 to 50 cm. This range was shorter than the previous study (Schenkman & Nilsson, 2010) that covered from 50 to 500 cm exceeding the peripersonal space range (Kolarik, Moore, Zahorik, Cirstea, & Pardhan, 2016). After the echolocation task was completed, we conducted a visual spatial working memory task (Luck & Vogel, 1997; Tsubomi, Fukuda, Watanabe, & Vogel, 2013) to evaluate the participants’ capacity for visual working memory.

## Methods

### Participants

In total, 39 sighted students from Hokkaido University (18–25 years; 17 females) participated in the experiment for monetary compensation or course credit. Three participants were excluded from the analyses due to failure to follow the instructions. One of the remaining 36 was left-handed. In echolocation task, the 36 participants completed the task on the first and second days, and 24 of the 36 (19–25 years; six females) participants completed the follow-up task on the third day. In visual working memory task, in total, the same 24 and 1 participant who did not participate the follow-up echolocation task completed the working memory task. One of the 25 was excluded from the analyses because of a high percentage of error responses (61.1%). All participants reported normal or corrected to normal visual acuity. None of the participants were hearing impaired according to self-report and a hearing test given before the main experiment. During the pretest, the participants were required to detect tones (500–8000 Hz, <20 dB HL) randomly played via headphones (MDR-XB450; Sony Co., Ltd., Tokyo, Japan) in the right and left ears separately. The intensity level of the playing tones was calibrated by a sound level meter (TM-103; Tenmars Electronics Co., Ltd., Taipei, Taiwan) and converted from dB SPL into HL using a conversion factor (ANSI S3.6-2010). None of the participants had prior experience with the echolocation task. This study was approved by the Ethics Board of Hokkaido University, and the participants provided written informed consent prior to the experiment.

### Apparatus and Stimuli

#### Echolocation task

**Figure 1.**
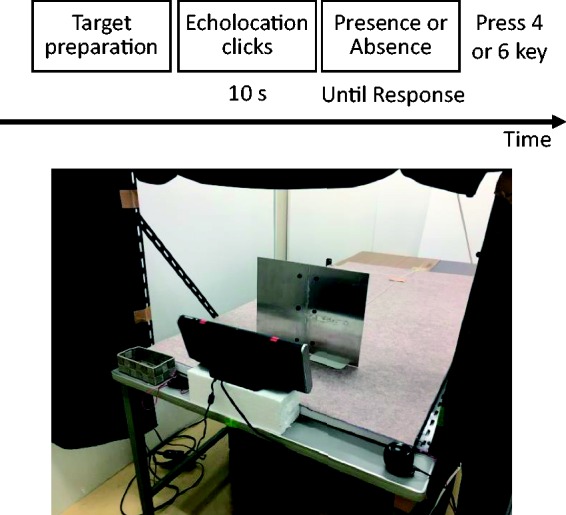
The object-detection task. Participants were seated in a chair and produced echolocation cues from a loud speaker by pressing a key on a numeric keypad to identify the presence/absence of a target.

Figure 1 shows the apparatus used for the detection task during the 3 days. The experiment was conducted in a space separated by a curtain (1.6 m width × 4.8 m depth × 2.5 m height) in a quiet laboratory. The wall was covered with 2 cm thick Styrofoam board to reduce noise reflection. The ceiling was covered with plasterboard. The ambient sound level in the space was approximately 34 dB as measured using a sound level meter. A table (1.1 m width × 2.4 m depth × 0.7 m height) was placed in the middle of the space. The top surface of the table was covered with carpet fabric placed on a 3 cm thick Styrofoam board. A loudspeaker (main speaker; SRS-BTX500; Sony Co., Ltd.; 20–20000 Hz), emitting echolocation cues, was mounted on the shorter edge of the table and was connected to a PC/AT-compatible computer (OptiPlex 990; Dell Inc., Round Rock, TX, USA) operating with Linux via a standard audio device (ALC269VB; Realtek Semiconductor Corp., Hsinchu, Taiwan). Another loudspeaker (sub speaker; SRS-BTV5; Sony Co., Ltd.; 20–20000 Hz) was mounted on the right side of the longer edge and was connected to an audio player (iPod Touch; Apple Inc., Cupertino, CA, USA). Two steel bars (2 mm thickness and 90 cm height) were placed vertically on the shorter side of the table and a black roll curtain was mounted on the top to block the participants’ vision. The participant was seated to the rear of the main speaker and the curtain on a height-adjustable chair, facing 3.6 m away from the front wall of the space and 0.85 m away from the nearest wall. Responses were collected via a numeric keypad connected to the computer. To send a signal to the participant, we used a vibration motor driven by the computer via a microcontroller board (Arduino Uno R3; BCMI US LLC, Boston, MA, USA).

The target was a flat aluminum plate (3 mm thickness; 40 cm width × 30 cm height) vertically mounted on an L-shaped bookend (1 mm thickness; 13 cm width × 17 cm height). The position of the target was experimentally manipulated (20, 30, 40, or 50 cm from the participant’s body).

**Figure 2.**
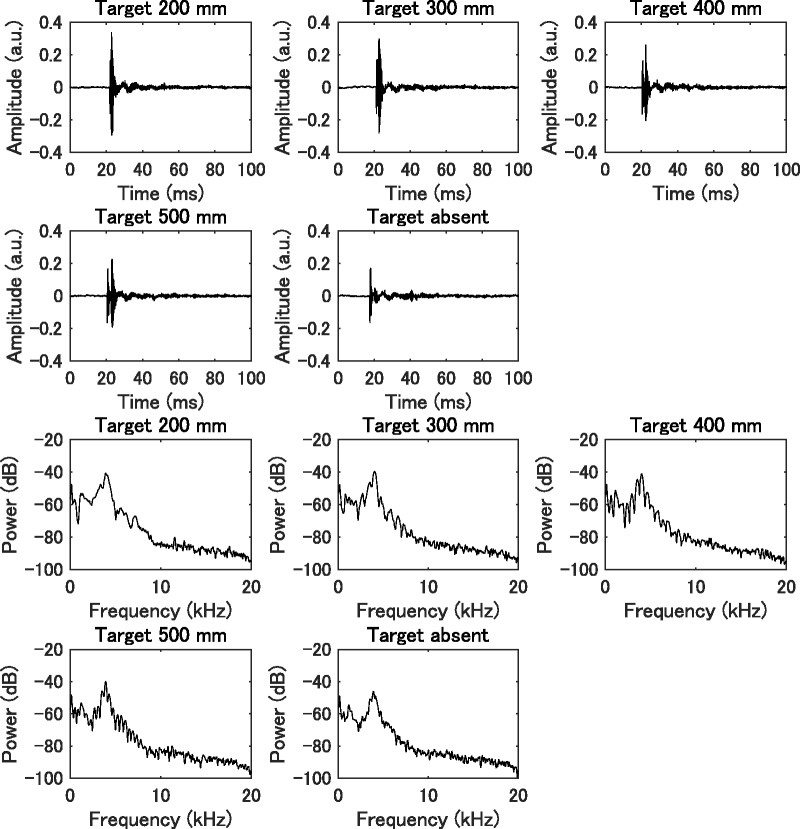
Waveform of a click produced through the loudspeaker (top half of figure) and the click’s power spectrum (bottom half of figure) for each target distance (20, 30, 40, or 50 cm) and for the target absent conditions.

The sound cues for echolocation were presented through the main speaker as approximately 6-ms-long artificial clicks generated by a Matlab function as a sinusoidal of exponentially decaying 4 kHz by 24-bit resolution and a 96 kHz sampling rate. We chose this function to simulate a waveform for a mouth click because it has been established that a sinusoid amplitude modulated by the exponentially decaying well represents human mouth clicks (Rojas, Hermosilla, Montero, & Espí, 2009; Thaler & Castillo-Serrano, 2016). One click was played through the main speaker at 95 dB when the participant pressed the “5” key on the numeric keypad. Each signal was captured with a microphone (B3 Omnidirectional Lavalier; Countryman Associates, Menlo Park, CA, USA; 20–20000 Hz) and digitized with 32-bit accuracy at a 96-kHz sampling rate using a high-speed USB audio interface (OCTA-CAPTURE; Roland, Shizuoka, Japan). Illustrations of the wave form and power spectrum are shown in Figure 2.

#### Visual spatial working memory task

**Figure 3.**
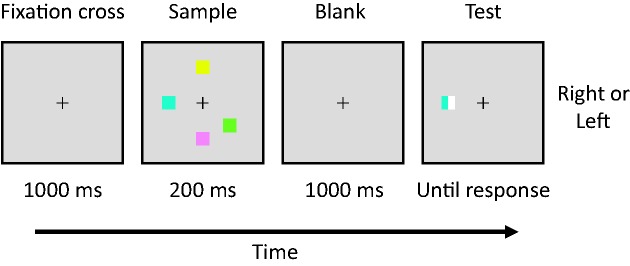
Schematic diagram of the visual spatial working memory task. Participants viewed a sample display for 200 ms followed by a test display. The participants were required to identify a color binding a location of a prior sample stimulus by providing a *right* or *left* response.

We used a standard visual spatial working memory task and colored squares as stimuli (Luck & Vogel, 1997; Tsubomi et al., 2013). An example of the stimuli is illustrated in Figure 3. Participants were required to memorize color–location combinations of the sample stimuli and to indicate whether the color of the cued patch in the test stimuli followed the sample stimuli separated by a blank display was identical to the color of the corresponding patch in the sample. The stimuli were displayed on a computer monitor (G2420HD; BenQ Co., Ltd., Haryana, India; driving at a rate of 60 Hz of refresh, 1920 × 1080 pixels), controlled by custom Matlab code using Psychophysics Toolbox extensions (Brainard, 1997; Pelli, 1997). The viewing distance was approximately 57 cm. Responses were collected via a computer keyboard.

All stimuli and test cues were displayed on a gray background on the monitor. A sample stimulus array consisted of colorful squares (four levels of memory set sizes: three, four, five, or six squares, 1° × 1° in width and height of the visual angle). Each square was randomly assigned one of a set of nine highly discriminable colors (red, pink, orange, yellow, green, yellow-green, blue, light blue, and white) without replacement and locations from a set of eight possible locations spaced equally 45° along an invisible circle of a radius of 2.9° in visual angle. A square cue (1° × 1° in width and height in visual angle) indicated one of the sample stimulus locations, so that the vertical halves of the area were filled with two different colors. One of the colors was the same as the sample at the cued location, and the other was a new color that was not presented in a sample array. Participants indicated the color they saw in the sample display.

### Procedure

#### Echolocation task

All participants completed the echolocation task over 2 days separated between 1 and 8 days (*M* = 2.83 day, *SD* = 2.23) and completed the task on the third day after 30 to 137 days (*M* = 77.3 day, *SD* = 42.2) from the second day. The participants did not repeat the task 2 or 3 times in the same day.

The experiment was a 3 × 4 × 2 factorial design with three within-subjects factors of day (1, 2, or 3), target distance (20, 30, 40, or 50 cm), and target presence (presence or absence). Each condition was randomly assigned for each trial. A session consisted of 32 practice trials followed by 120 test trials per day. One participant quit the test at 80 trials due to a technical error. The trials were completed in approximately 70 minutes per day, including 15-minute practice trials and one 5-minute break.

All participants were visually impeded by a close-fitting eye mask at the beginning of the trials. The participants fixed their head position at approximately 10 to 20 cm above the center of, and 5 to 15 cm behind, the main speaker; thus, their head was not hidden by it. We did not use a chinrest to avoid discomfort to the participants caused by a long period of physical restraint. Each participant held the vibration motor by their nondominant hand or placed it on their thighs. To mask sounds related to the placement of the target, the black roll curtain obstructed the target from the participant and the subspeaker played 80 dB of pink noise until the target was placed on the table (or was not placed, in the target-absent trials). After the target was ready, the participant was prompted by the vibration motor signal and allowed to produce clicks for 10 seconds by pressing the “5” key on the numeric keypad with their dominant hand. Another vibration signaled the end of the 10 seconds of the trial, and the participant pressed the “6” key on the keypad for presence or the “4” key for absence. The responses and number of clicks were recorded by the computer. We calculated the discriminability index *d* ′ (Green & Swets, 1974) as the difference between the *z*-transforms of the hit and false alarm rate; *d* ′ = *z* (hit rate) – *z* (false alarm rate). The hit and false alarm rates were converted from 1 to 1 – 1/(2*N*) and from 0 to 1/(2*N*) (Macmillan & Creelman, 1991).

Participants received feedback by vibration upon providing a correct answer for target presence/absence during the practice trials to familiarize themselves with detection task procedure. However, they received no such feedback during the experimental trials.

#### Visual spatial working memory task

The visual working memory capacity task was conducted on the first or third day of the echolocation task depending on each participant’s availability. Each participant performed a total of 216 trials consisting of 54 trials per condition (three, four, five, or six of the memory set size). As shown in Figure 2, each trial began with the presentation of a central fixation cross (0.2° × 0.2°) for 1,000 ms, followed by the sample stimulus array for 200 ms. After a blank period of 1,000 ms, the test cue remained on the screen until the participant responded. The participants indicated whether the cued color was on the right or left by pressing the keys for “i” (for right) or “e” (for left) responses. We computed the estimating capacity score of visual working memory with the common formula: *K* = *S* (*P* – 50)/50, where *K* is the visual capacity, *S* is the memory set size, and *P* is percentage correct (Cowan, 2001). The formula represents the individual’s ability to hold *K* items from a sample array of *S* items in their working memory.

## Results

### Detection Performance Across 2 Days

**Figure 4.**
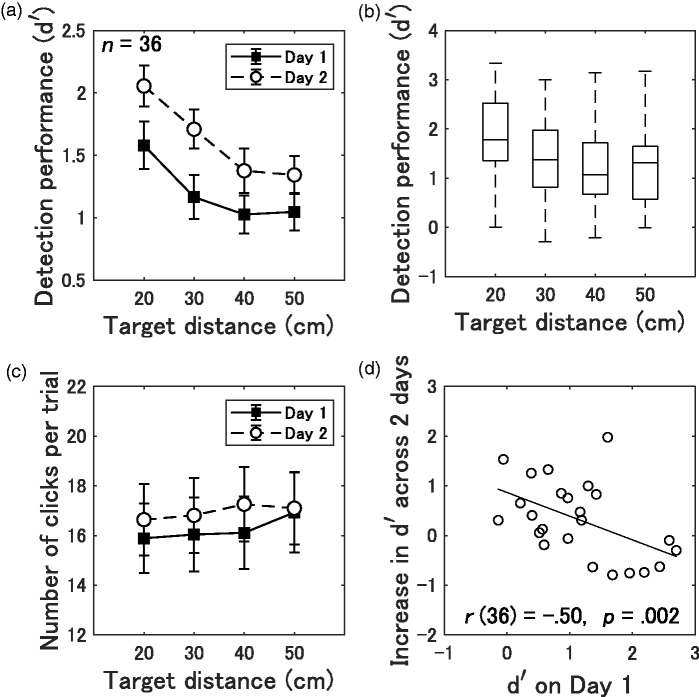
(a) Mean *d* ′ score for Day 1 or 2 as a function of target distance (20, 30, 40, or 50 cm). Error bars represent SEMs. (b) Box-and-whisker plots showing the median, interquartile, and range with 1.5 × interquartile range whiskers for the *d* ′ score averaged across the 2 test days. (c) Mean number of clicks for Day 1 or 2 as a function of target distance (20, 30, 40, or 50 cm). Error bars represent SEMs. (d) Plots of the increase in *d* ′ score averaged across the 2 test days as a function of *d* ′ on Day 1. The solid line represents the regression line of the plot.

The *d* ′ values obtained from individual participants (*n* = 36) were averaged separately for each target distance. Figure 4(a) shows the detection performance of the echolocation task on the first and second days. To test the improvement in detection performance across the 2 days, we subjected the *d* ′ scores to a 2 (Day 1 and 2) × 4 (Target distance 20, 30, 40, and 50 cm) repeated-measures analysis of variance (ANOVA). The ANOVA revealed significant main effect of day, *F*(1, 35) = 9.22, *p* = .005, ${\text{η}}_{\text{p}}^{\text{2}}$ = .21, and target distance, *F*(3, 105) = 17.27, *p* < .001, ${\text{η}}_{\text{p}}^{\text{2}}$ = .33. Their interaction was not significant, *F*(3, 105) = 0.80, *p* = .494, ${\text{η}}_{\text{p}}^{\text{2}}$ =.02. Multiple comparisons by Holm’s method revealed that the *d* ′ score decreased from 20 to 30 cm, *t*(35) = 3.92, *p* = .002, *r* = .55; from 20 to 40 cm, *t*(35) = 6.54, *p* < .001, *r* =.74; and from 20 to 50 cm, *t*(35) = 5.49, *p* < .001, *r* = .68.

The *d* ′ score averaged across both days fluctuated substantially within participants (boxplot, Figure 4(b)); the means (*SD*) were 1.82 (0.87), 1.44 (0.85), 1.20 (0.84), and 1.20 (0.81) when the target was located at 20, 30, 40, and 50 cm, respectively. These scores were significantly higher than 0 when the target was placed at 20 cm, *t*(35) = 12.59, *p* < .001, *r* = .91; at 30 cm, *t*(35) = 10.11, *p* < .001, *r* = .86; at 40 cm, *t*(35) = 8.62, *p* < .001, *r* = .82; and at 50 cm, *t*(35) = 8.87, *p* < .001, *r* = .83.

We also investigated the ceiling effect for improvement in detection performance. Participants who performed well on Day 1 improved less than those whose initial performance was poorer. The increase in detection performance was calculated by subtracting the *d* ′ of Day 1 from the *d* ′ of Day 2. Figure 4(c) shows a plot of the increase in *d* ′ score as a function of *d* ′ on Day 1. We computed Pearson’s correlation coefficients, and the results showed a significant negative correlation between the observed increase in performance and *d* ′ score on Day 1, *r*(36) = −.50, *p* = .002, indicating that high-performing individuals improved their performance less across the 2 days than did low-performance individuals.

We also assessed the effects of increasing in the number of clicks during the detection task. The participants’ number of emission sounds per trial are shown in Figure 4(d), as a function of target distances during the target trials. The mean number of clicks per trial was subjected to a repeated-measures ANOVA with a 2 (Day 1 and 2) × 4 (Target distance 20, 30, 40, and 50 cm) design. The results revealed a significant effect of target distance, *F*(3, 105) = 2.96, *p* = .036, ${\text{η}}_{\text{p}}^{\text{2}}$ =.08; while a no significant effect of day, *F*(1, 35) = 0.68, *p* = .414, ${\text{η}}_{\text{p}}^{\text{2}}$ = .02, and their interaction, *F*(3, 105) = 1.54, *p* =.210, ${\text{η}}_{\text{p}}^{\text{2}}$ =.04. There was no significant correlation between the mean number of clicks per trial averaged across the distances and the *d* ′ score, *r*(36) =.09, *p* = .607; this indicated that an increase in the number of clicks did not improve or impair detection performance.

### Detection Performance on the Third Day

**Figure 5.**
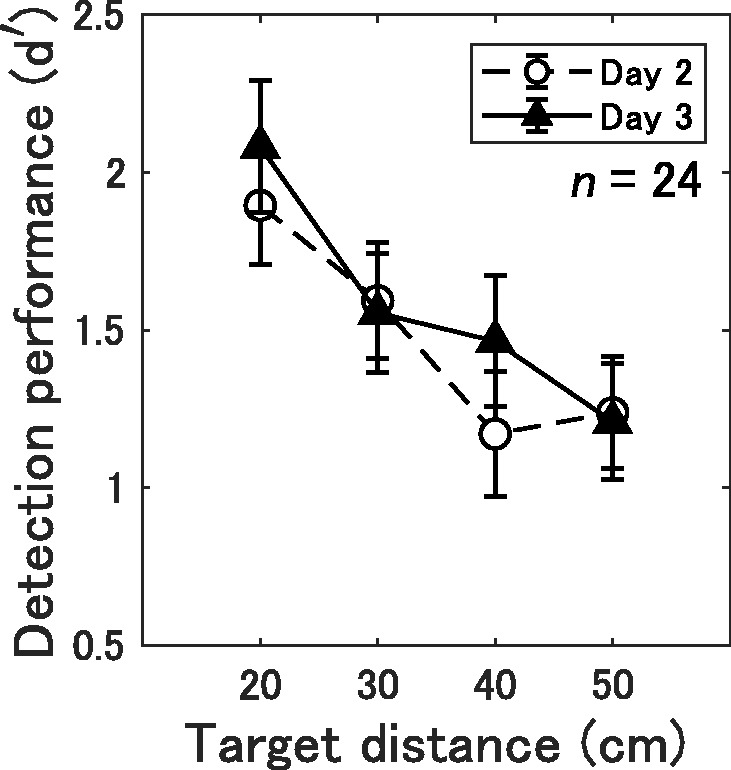
Mean *d* ′ score of the 24 samples for Day 2 or 3 as a function of target distance (20, 30, 40, or 50 cm). Error bars represent SEMs.

The *d* ′ on Day 3 obtained from individual participants were averaged separately for each target distance. Figure 5 shows the detection performance of the echolocation task on Days 2 and 3 in the 24 samples who completed the follow-up echolocation task on the third day. To examine maintaining improved detection performance over a month, we subjected the *d* ′ scores to a repeated-measures ANOVA with a 3 (Day 2 and 3) × 4 (Target distance 20, 30, 40, and 50 cm) design. The ANOVA revealed significant main effect of target distance, *F*(3, 69) = 16.34, *p* < .001, ${\text{η}}_{\text{p}}^{\text{2}}$ = .42; while no significant effect of day, *F*(1, 23) = 0.56, *p* = .461, ${\text{η}}_{\text{p}}^{\text{2}}$ = .02, and their interaction, *F*(6, 69) = 1.50, *p* = .222, ${\text{η}}_{\text{p}}^{\text{2}}$ =.06, which indicated that improved detection performance was retained for at least 1 month.

### Visual Working Memory Capacity

**Figure 6.**
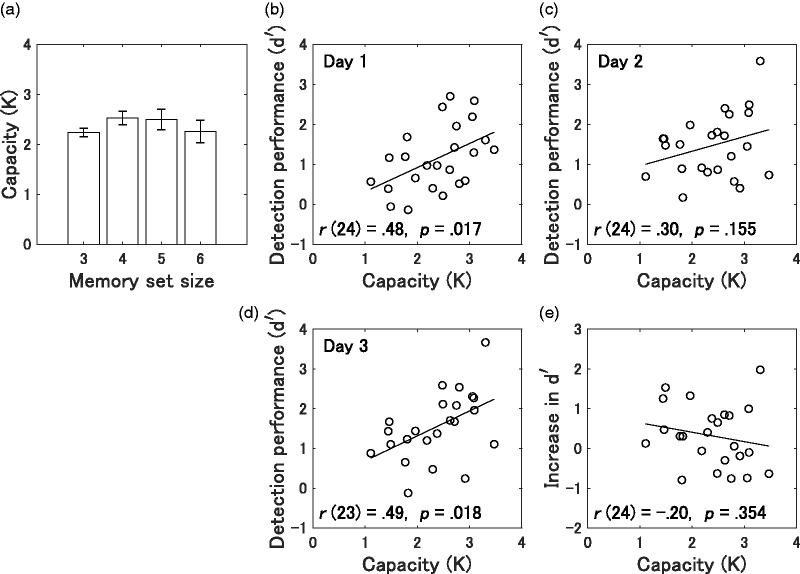
Behavioral results of the visual working memory task. (a) Mean visual working memory capacity (*K*) as a function of memory set size (3, 4, 5, or 6). Error bars represent SEMs. (b–d) Correlations between visual working memory capacity and *d* ′ for Day 1, Day 2, or Day 3. It should be noted that several points overlapped each other. The solid line represents the regression line of the plot. (e) Correlation between visual working memory and increase in *d* ′ across the 2 test days.

We examined the association between visual working memory capacity and object detection performance by echolocation, separately for each experimental day (Days 1–3). We also examined whether improved detection performance across 2 days was modulated by working memory capacity. Plots of detection performance scores as a function of the capacity *K* are shown in Figure 6. The mean performance (*K*) on the task was 2.38 (95% CI [2.10, 2.65], *SD* = 0.65). The results revealed a significant positive correlation between the visual working memory capacity score and the *d* ′ score on Day 1, *r*(24) = .48, *p* = .017; and on Day 3, *r*(23) = .49, *p* = .018; there was no significant correlation between visual working memory capacity score and either the detection performance on Day 2, *r*(24) = .30, *p* = .155, or the increase in performance, *r*(24) = −.20, *p* = .354.

## Supplementary Experiment

To examine whether visual working memory capacity modulated detection performance on echolocation tasks, we further measured each individual’s visual cognitive performance using a matrix test (Cornoldi, Cortesi, & Preti, 1991), the score of which can be taken as a measure of spatial working memory capacity. In this test, participants were asked to follow an imaginary pathway through two-dimensional matrices. Twelve of the 36 original participants performed the matrix test. In addition, five students who did not perform the initial echolocation task participated in the same echolocation task for only 1 day as well as in the matrix test. Total of 17 participants (18–26 years; 7 females) completed the matrix test on the second day of the experiment or the earlier, depending on each participant’s availability. We examined the correlation between the performances on the matrix test and on echolocation task on the first day.

**Figure 7.**
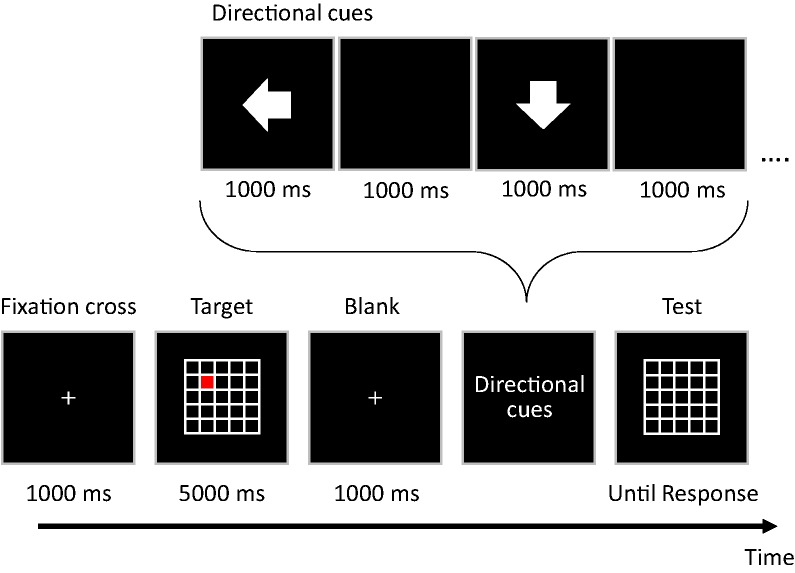
Schematic diagram of the matrix test. Participants followed an imaginary pathway through two-dimensional matrices (11 × 11; the example shows a scaled down 5 × 5 grid). The participants were required to identify the final destination of the target.

Figure 7 shows a schematic diagram of a trial of the matrix test. All stimuli were displayed on a black background on an LCD monitor (XL2411T; BenQ; 100 Hz refresh rate, 1,920 × 1,080 pixels). Participants were required to memorize a target location displayed in a cell of an 11 × 11 matrix, subtending 24° × 24° in width and height of the visual angle. Their task was to mentally maneuver the target according to visual directional cues. Each trial began with the presentation of a central fixation cross (0.2° × 0.2°) for 1,000 ms, followed by the target stimulus in the matrix for 5,000 ms. The target was a red square, the initial location of which was randomly selected from the matrix. After a blank screen was shown for 1,000 ms, arrow cues (9° × 9°) were sequentially presented in the center of the screen. The set size of the series of the cues (4, 6, or 8) and direction (right, left, up, or down) were assigned randomly. Each directional cue was presented for 1,000 ms, and the cues were separated by a blank screen presented for 1,000 ms. After the presentation of a series of directional cues, the matrix was displayed on its own. Participants indicated the final target destination by clicking a cell in the matrix. Each participant performed a total of 30 trials consisting of 10 trials of each set size of the sequence length.

**Figure 8.**
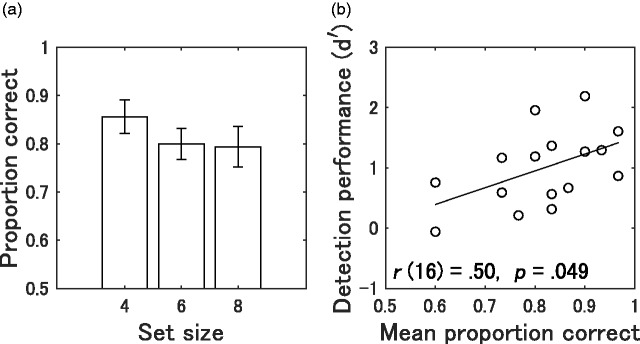
Behavioral results of the matrix test. (a) Mean proportion of correct responses as a function of memory set size (4, 6, or 8). Error bars represent SEMs. (b) Correlation between the proportion of correct response and echolocation performance (*d* ′) on Day 1. The solid line represents the regression line of the plot.

The proportions of correct responses obtained from individual participants were averaged across the sequence length. The mean correct proportion was 0.82 (95% CI [0.76, 0.88], *SD* = 0.11). One participant was removed from the analysis as an outlier, indicated by a Cook’s distance exceeding 0.5. We examined the association between the mean proportion of correct responses and echolocation performance on the first day (*n* = 16). The plot of the *d* ′ scores on Day 1 against the mean proportion of correct responses is shown in Figure 8. We computed Pearson’s correlation coefficients, and the results revealed a significant positive correlation between the matrix test score and the *d* ′ score on Day 1, *r*(16) = .50, *p* = .049.

## Discussion

We examined the association between echolocation performance in sighted individuals and individual factors, such as visual working memory capacity. We found that the participants’ visual working memory capacity was positively correlated with detection performance, expect on the second day. Detection performance did not decline over several months. In other words, we found that the improved detection achieved on the second day was maintained for at least 30 to 137 days.

Interestingly, detection performance improved over the localization task repeated for 120 trials (approximately 50 minutes) per day without any feedback, although participants did not perform as well as subjects in a previous study that included feedback on every trial (Schenkman & Nilsson, 2010). This indicates that sighted participants can improve their performance following a relatively small amount of repetitive training during the task. However, we found individual differences in detection performance. Specifically, individuals’ performances on target detection tests fluctuated substantially, reflected by the large standard deviations and range values of the data.

The present results extend the findings of Ekkel et al. (2017), in that we assessed spatial working memory capacity; participants required to memorize visual objects being actively used during visual spatial processing while not involving auditory verbal processing. Our results show a significant correlation between visual working memory capacity and detection performance during echolocation, except for performance on the second day, after performance had improved. The correlation between visual working memory capacity and performance on the second day was not significant, and the coefficient value decreased compared to the first day. This lack of a correlation on the second day might be due to the improvement in performance. Individuals who performed well on the first day of the tests improved less than those who initially had low scores, and thus the regression line of detection performance on working memory capacity flattened out on the second day, decreasing the correlation coefficient value.

Our results were inconsistent with those of by Ekkel et al. (2017), in that we did not find a significant correlation between working memory capacity and the increase in detection performance across the 2 days. This difference seems reasonable because of the ceiling effect for enhancement of echolocation ability. Specifically, because there was a positive correlation between echolocation performance and working memory capacity on the first day, individuals who had large memory capacities were expected to show a smaller increase in detection performance across the 2 days. In fact, the results indicated a negative (but nonsignificant) correlation (*r* = −.20). Another possible explanation is that the study by Ekkel et al. (2017) assessed participants’ working memory capacity in the context of the central executive component, rather than a spatial subcomponent, whereas this study measured the working memory capacity as a reflection of spatial aspects. Also, Ekkel et al. assessed performance using a size discrimination task, whereas this study used a different type of task, involving object detection (Schenkman & Nilsson, 2010). Additional research is needed to determine the influence of types of echolocation and working memory tasks on correlations between working memory capacity and echolocation ability.

In summary, visual working memory capacity predicts object detection performance on an echolocation task. The present finding adds to the literature in that the correlation between echolocation ability, rather than the degree of improvement in echolocation due to training, and visual working memory capacity has never been examined. The involvement of vision during echolocation processing has been suggested by Thaler et al. (2014), who indicated that echolocation involves spatial cognitive processing (e.g., mental imagery). The visual area may not be involved during other auditory control tasks (e.g., change in the sound location; Thaler & Foresteire, 2017). The present results support these findings regarding visual involvement in echolocation and the spatial component of working memory by sighted participants. Furthermore, the findings suggest that visual working memory capacity is predictive of higher performance of the detection task for sighted participants. The question of how much training is necessary to learn echolocation remains unresolved. To clarify this issue, future studies should manipulate the amount of echolocation task training to determine a sufficient amount of training as well as to investigate how performance improves.
